# Redundancy Analysis to Reduce the High-Dimensional Near-Infrared Spectral Information to Improve the Authentication of Olive Oil

**DOI:** 10.1021/acs.jcim.2c00964

**Published:** 2022-09-21

**Authors:** María Isabel Sánchez-Rodríguez, Elena Sánchez-López, Alberto Marinas, José María Caridad, Francisco José Urbano

**Affiliations:** † Department of Statistics and Business, Faculty of Law and Business, University of Cordoba, Avda. Puerta Nueva, s/n., E-14071 Cordoba, Spain; ‡ Department of Organic Chemistry, University of Cordoba, Campus de Rabanales, Marie Curie Building, E-14014 Cordoba, Spain

## Abstract

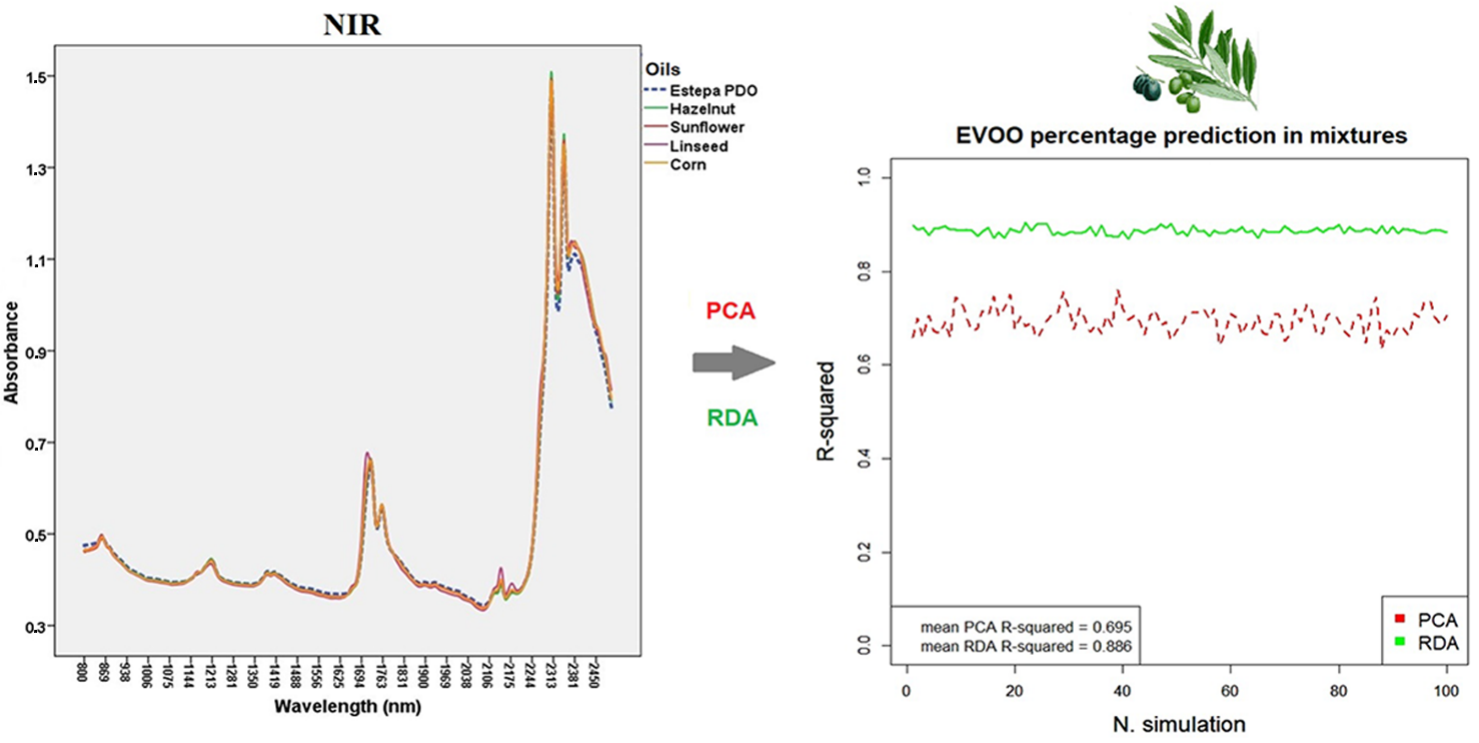

The high price of marketing of extra virgin olive oil (EVOO) requires the introduction of cost-effective and sustainable procedures that facilitate its authentication, avoiding fraud in the sector. Contrary to classical techniques (such as chromatography), near-infrared (NIR) spectroscopy does not need derivatization of the sample with proper integration of separated peaks and is more reliable, rapid, and cost-effective. In this work, principal component analysis (PCA) and then redundancy analysis (RDA)—which can be seen as a constrained version of PCA—are used to summarize the high-dimensional NIR spectral information. Then PCA and RDA factors are contemplated as explanatory variables in models to authenticate oils from qualitative or quantitative analysis, in particular, in the prediction of the percentage of EVOO in blended oils or in the classification of EVOO or other vegetable oils (sunflower, hazelnut, corn, or linseed oil) by the use of some machine learning algorithms. As a conclusion, the results highlight the potential of RDA factors in prediction and classification because they appreciably improve the results obtained from PCA factors in calibration and validation.

## Introduction

Extra virgin olive oil (EVOO) is an excellent edible oil that is greatly prized for its taste and its beneficial health properties.^1,2^ It consists of an *Olea europaea* juice obtained by mechanical procedures only, thus maintaining all of their favorable characteristics. EVOO is formed by a complex matrix of chemical compounds of two main groups. The saponifiable fraction or major component consists of fatty acids that usually form esters, most often with glycerol, to produce glycerides (mono-, di-, and triacylglycerols) and phosphatides and some free fatty acids. The fatty acids are classified as saturated fatty acids (SAFAs), including palmitic, margaric, stearic, and arachidic acids; monounsaturated fatty acids (MUFAs), including palmitoleic, margaroleic, oleic, vaccenic, and gadoleic acids; and polyunsaturated fatty acids (PUFAs), including linoleic and linolenic acid. Oleic acid is the fatty acid present in the highest percentage (55–85%) and provides EVOO with a monounsaturated oil character, in contrast with other cheaper oils extracted from oilseeds that are richer in PUFAs. The unsaponifiable fraction or minor component consists of a heterogeneous group of chemicals such as sterols, fatty alcohols, pigments (chlorophylls and carotenoids), tocopherols and tocotrienols, and volatile and phenolic compounds.

Due to its high quality, EVOO is subject to fraud in its marketing, like adulterations with lower-quality oils (halzenut, sunflower, linseed, palm, and corn oils, among others). For this reason, the establishment of procedures to achieve reliable authentication of EVOO is essential. Classical techniques such as chromatography (gas and liquid) for separation coupled to other identification methods are widely used to determine the traceability of olive oil and provide some well-resolved information.^3−5^ Therefore, for instance, the study of the fatty acid profile (by gas chromatography (GC) as fatty acid methyl esters (FAMEs)) can lead to the detection of adulterations of virgin olive oils with deodorized^6^ and other vegetable^3^ oils. However, chromatographic techniques are slow and expensive and require sample preparation and the use of solvents. On the other hand, vibrational spectroscopic analytical techniques (e.g., near-infrared (NIR), mid-infrared (MIR), or Raman spectroscopy) are also widely used in the determination of olive oil adulterations.^7−12^ These techniques are fast, cost-effective, and on-line and allow comprehensive analysis of the olive oil since it is formed by a complex matrix of chemicals, but they provide continuous information with overlapping bands and signals that are not as clearly resolved as in the case of conventional techniques. Therefore, the application of chemometrics tools is required to extract some important quantitative or qualitative information related to the authentication of EVOO.^11−15^

Moreover, principal component analysis (PCA) is used in linear modeling to synthesize high-dimensional information. This is the case for NIR spectra of oils, where the number of explanatory variables greatly exceeds the number of observations. PCA calculates linear combinations of the explanatory variables by maximizing their variability. Redundancy analysis (RDA) can be seen as a constrained version of PCA: given two groups of variables, RDA searches for linear combinations of variables in one group that maximize the variance of the other group that is explained by each one of the linear combinations. RDA has been recently applied and developed in works on different issues such as health,^16,17^ biology,^18^ and the environment.^19^ Moreover, some recent works^9,12,20,21^ have used PCA to reduce NIR spectral information to classify oils from different machine learning algorithms (e.g., linear discriminant analysis (LDA) or support vector machine (SVM)). However, the literature does not include applications of RDA to reduce the high dimensionality of NIR spectra with the objective of predicting a numerical or non-numerical variable (e.g., the content of any compound or the type of vegetable oil, respectively) to improve the authentication of EVOO.

Therefore, the aim of this study is to use both PCA and RDA to reduce the high-dimensional NIR spectral information on oils. The corresponding PCA and RDA factors are included as explanatory variables, first in regression models to predict the percentage of EVOO in blended oils and second in machine learning classification models to estimate the type of pure vegetable oil or the class of adulterated mixture. The quantitative and qualitative results are compared in terms of calibration and validation, that is, taking into account estimation errors from data used or not used in the modeling. As a conclusion, RDA factors provide the best results in regression and classification, and therefore, their potential in the authentication of EVOO is highlighted.

In particular, the structure of the paper is the following. The next section describes the data acquisition process (for GC and NIR data) and the statistical methodology. Next, the results are presented and discussed: first, PCA and RDA are used for NIR dimensionality reduction; second, PCA and RDA factors are considered as explanatory variables in quantitative models for EVOO percentage estimation and in qualitative models for predicting the pure vegetable oil or, for a certain vegetable oil, the type of mixture with EVOO. Finally, the last section includes the main conclusions of the work and some future research lines.

## Materials and Methods

### Samples

The study includes 30 EVOO samples with a Protected Designation of Origin (PDO)—particularly, from the Andalusian Estepa (Sevilla) PDO—with the aim to ensure its purity. In addition, 480 oils were obtained from adulteration or mixture of EVOO with four lower-quality vegetable oils (refined hazelnut, sunflower, linseed, or corn oil purchased from stores) at six levels: 2, 10, 15, 25, 50, and 75 wt % (20 samples of each vegetable oil and percentage of adulteration). The low percentages of adulteration were selected to represent the type of real adulteration found in the market. All levels of adulteration discussed are listed as weight percentages as described in the following equation:
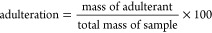
where the total mass of sample was 5 g.

The mixtures were shaken vigorously to ensure complete homogenization, and all of the samples were bottled in amber glass flasks and maintained in the dark at 2 °C until analysis. Finally, 80 samples corresponded to pure refined hazelnut, sunflower, linseed, or corn oil (20 samples of each type).

### Gas Chromatography

The determination of fatty acid composition by GC was carried out according to official methods for controlling olive and pomace oil stabilized by the European Union Commission^22^ and the International Olive Oil Council.^23,24^ An Agilent 7890A gas chromatograph with a capillary column (SGE BPX-70 FORTE 50 m × 220 μm × 0.25 μm) was used. A flame ionization detector (FID) was used, as it is one of the most used and versatile. The analysis conditions were as follows: the inlet temperature was 250 °C; the injection volume was 2 μL; the detector temperature was 260 °C; and the oven temperature was programmed to remain at 180 °C for 15 min and then raised to 240 °C at a rate of 4 °C/min and maintained at this temperature for 5 min. Analyses were carried out in triplicate using the average values in the statistical study. Oil samples were initially subjected to a cold transesterification process to convert triacylglycerols into FAMEs. This method is intended for edible oils with acidity index lower than 3.3°. In this process, 0.1 g of oil was transferred to a 5 mL volumetric flask, and then 2 mL of *n*-heptane and 0.2 mL of 2 N KOH in methanol were added. The mixture was vigorously stirred, and then the methyl esters were extracted and subjected to analysis by GC. The oil composition in terms of SAFAs, MUFAs, and PUFAs was determined from the corresponding GC chromatogram. These values were considered as a reference for statistical studies.

### NIR Spectra

**Figure 1:**
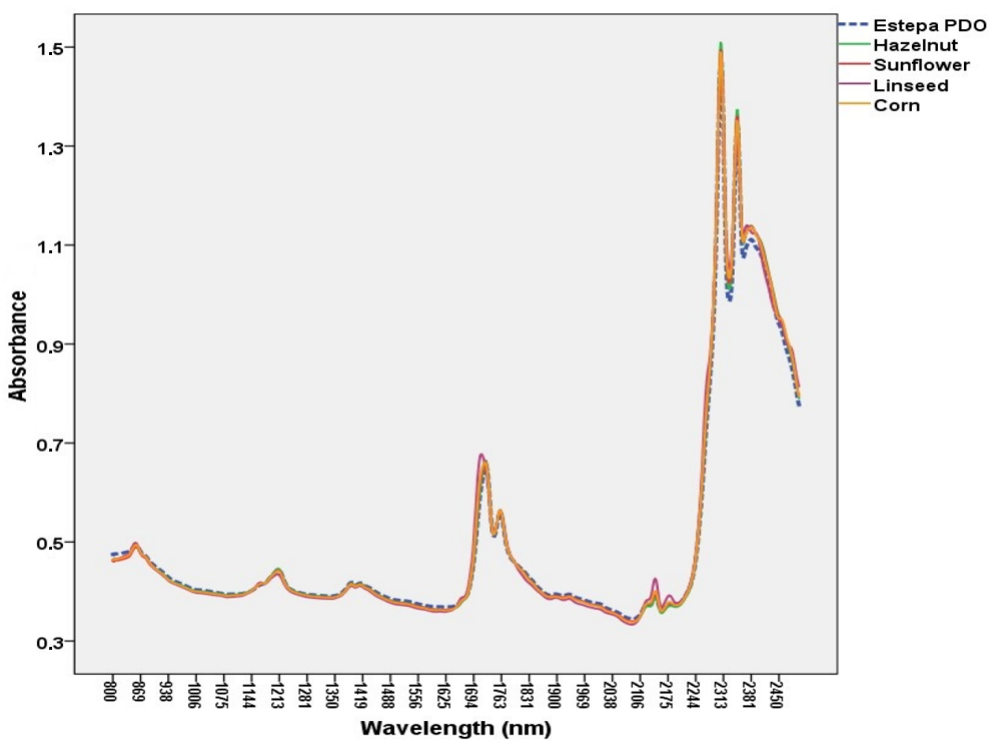
NIR spectra of EVOO and other vegetable oils.

Transflectance NIR spectra were collected with a NTS Spectrum One FT-NIR spectrophotometer (PerkinElmer LLC, Shelton, CT, USA) equipped with an integrating sphere module, available at the Central Service of Research Support (SCAI) of the University of Cordoba. The analysis was carried out not later than 15 days from the date of receipt of samples, which were stored in the refrigerator at 4 °C so that their properties were not altered.^25^ Spectra were obtained using Spectrum Software 5.0.1 (PerkinElmer LLC). The reflectance (log 1/*R*) spectra were collected with two different reflectors with the same material and shape to avoid measurement errors and rule out variability. Besides, spectra were smoothed by the technique of Savitzky and Golay,^26^ which applies local polynomial least-squares regression to avoid instrumental random noise. Finally, the first derivatives of the spectra were obtained to avoid the effect of baseline deviation. Once pretreated, 1237 NIR data associated with measurements for each case (which represent the energy absorbed by each sample for each of the 1237 wavelengths from 800.62 to 2499.64 nm) were provided. Typical NIR spectra of an EVOO and other vegetable oils are depicted in Figure 1.

### Statistical Methodology

As stated above, *principal component analysis* is a widely used in statistical linear models with a number of explanatory variables that greatly exceeds the number of observations (as in the case of spectral NIR data). From the explanatory variables, PCA calculates a reduced number of orthogonal components or latent factors that summarize the information in the data. These PCA factors maximize the variance among the explanatory variables. Usually, a reduced number of PCA factors are enough to explain a high percentage of the variability of the data and thus prevent overfitting of the model. The objective is not only to summarize the information in a few components but fundamentally to use those components as explanatory variables in quantitative or qualitative statistical models.

Moreover, let *y*_1_, *y*_2_, ..., *y*_*n*_ and let *ŷ*_1_, *ŷ*_2_, ..., *ŷ*_*n*_ be the observations of a numerical dependent variable *Y* and the predictions from a regression model, respectively, and let *s*_*Y*_^2^ and *s*_*Ŷ*_^2^ be the corresponding variances. The coefficient of determination, *R*^2^ = *s*_*Ŷ*_^2^/*s*_*Y*_^2^, ranges in the interval [0, 1] and evaluates the goodness of fit of the model, which is better as *R*^2^ approaches 1. Specifically, this coefficient measures the calibration or training capability of the model, as it is calculated from data used for the estimation of the model. Given the predictions for the future *t* observations of the regression model, *ŷ*_*n*+1_, *ŷ*_*n*+2_, ..., *ŷ*_*n*+*t*_, the mean square error of the prediction, MSEP = ∑_*j*=1_^*t*^(*y*_*n*+*j*_ – *ŷ*_*n*+*j*_)^2^/*t*, evaluates its prediction (test or validation) capability. As the MSEP depends on the squared measurement units of *Y*, the following dimensionless expression is defined: DRMSEP = RMSEP/, where RMSEP is the square root of the MSEP and  is the average of the observations *ŷ*_*n*+1_, *ŷ*_*n*+2_, ..., *ŷ*_*n*+*t*_. The test capability of a model is obviously better as DRMSEP approaches 0. Besides, *R*^2^ and DRMSEP can be cross-validated by simulation, that is, by the design of an algorithm that modifies the partition of the original data set into calibration and validation data subsets at each iteration.

Furthermore, canonical redundancy analysis (RDA) is a technique of multivariate analysis where a matrix of response variables **Y** is explained by a matrix of explanatory variables **X**. Linearity and variance homogeneity between the variables of matrices **X** and **Y** are the main assumptions. Once **X** and **Y** are standardized to avoid the effect of the measurement units, RDA is developed in two steps: (*i*) multivariate regression of **Y** on **X**, which produces a matrix of fitted values **Ŷ**, and (*ii*) PCA of **Ŷ** to reduce its dimension in the called RDA components or *redundancy axes*. Each eigenvalue of the correlation matrix of the variables of **Ŷ**, λ_*j*_ for *j* = 1, ..., *g*, represents the variance of the corresponding redundancy axis. The goodness of fit of the technique is measured by the following quotient, called the *redundancy index*:
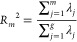
where *m* is the number of the first redundancy axes (among the possible *g* RDA components) to retain.^a^ The interpretation of the redundancy index is similar to that of a coefficient of determination: the reliability of the analysis is higher as the redundancy index approaches 1. As a result, matrices **X** and **Y** are represented in the two- or three-dimensional space formed by the first RDA factors. In this representation, the variables or cases with the highest scores (coordinates) are used to interpret the axes and show which variables or cases are discriminated by RDA. On the contrary, the proximity between variables or cases represents the high association between them. RDA is an alternative to canonical correlation analysis (CCA) presented by authors such as Rao^27^ and Van de Wollenberg;^28^ more recently, Legendre et al.^29^ tested the significance of the redundancy axes in RDA.

Among the machine learning classification procedures (i.e., in the case in which the dependent variable is qualitative or non-numeric) from chemical data,^30,31^ this study applies supervised classification, as the grouping into classes is previously known. First, *linear discriminant analysis* (LDA) predicts the membership of data to several a priori-defined classes. The discriminant functions are given as linear combinations of the explanatory variables, and their discrimination power can be measured by their corresponding canonical correlation, that is, the square root of the ratio between the intergroup sum of squares and the total sum of squares. Second, in *classification and regression trees* (CARTs), the task of data mining for the class estimation is built on particular characteristics of the data set. The procedure recursively partitions the data set and fits a simple prediction model within each partition with the aim of detecting what attribute or characteristic is the best forecaster for the accurate calculation of the problem, with prediction error measured in terms of misclassification cost. In the third place, *k nearest neighbor* (KNN) is a nonparametric and nonlinear technique for pattern recognition statistical estimation. The algorithm assigns each new case to the class most common among the plurality vote of its *k* nearest neighbors. The assignment is based on a distance function. The appropriate distance to use depends on the type of the classified variable, with the Hamming distance being the one used for non-numerical variables. Then, in *support vector machine* (SVM), each data point is viewed as a *p*-dimensional vector, and the aim is to separate such points with a (*p* – 1)-dimensional hyperplane. The best separation is achieved by the hyperplane that has the largest distance to the nearest training data point of any class and is associated in general with the lowest global error of classification. Finally, *random forest* (RF) is a combination of tree predictors such that each tree depends on the values of a random vector sampled independently and with the same distribution. The generalization error depends on the strength of the individual trees in the forest and the correlation between them. Internal estimates are also used to measure variable importance.

All of the previous machine learning classification algorithms are evaluated by using the *accuracy*, that is, the percentage of correctly classified instances out of all instances. Besides, another metric is also considered, *Kappa*, a measure similar to classification accuracy except that it is normalized at the baseline of random chance on the data set and it is more appropriate for problems with an imbalance in the classes. Accuracy and Kappa^b^ were calculated for calibration and validation, that is, taking into account data used or not used in the modeling.

As for the software, the R packages “pls”,^32^ “vegan”,^33^ and “caret”^34^ were used to obtain the PCA factors from NIR spectra and develop the multivariate analysis of RDA and machine learning classification. Detailed information on the code of the programs can be seen in the Supporting Information.

## Results and Discussion

The authentication of EVOO requires the application of fast, reliable, and cost-effective analytical procedures with no or little sample manipulation, such as NIR spectroscopy. Besides, NIR spectroscopy can be considered a green technology because it involves a low environmental impact.^35^ The assignment of bands in the NIR spectrum is straightforward, but the application of chemometrics is required to extract the maximum relevant information from the spectrum.

### PCA for NIR Dimensionality Reduction

First, the large amount of information contained in the NIR spectra is summarized by the use of PCA analysis. To prevent overfitting and to take into account some recommendations in the literature, such as the usual Kaiser criterion, three PCA factors were retained in this study: the proportions of explained variance for PCA1, PCA2, and PCA3 were 61.82, 30.32, and 6.26%, respectively (98.40% in total), and the eigenvalue associated with PCA4 was less than 1. Besides, as has been proved, the capability of the following analyses was not improved increasing the number of retained factors.

### RDA for Visualization and NIR Dimensionality Reduction

**Figure 2:**
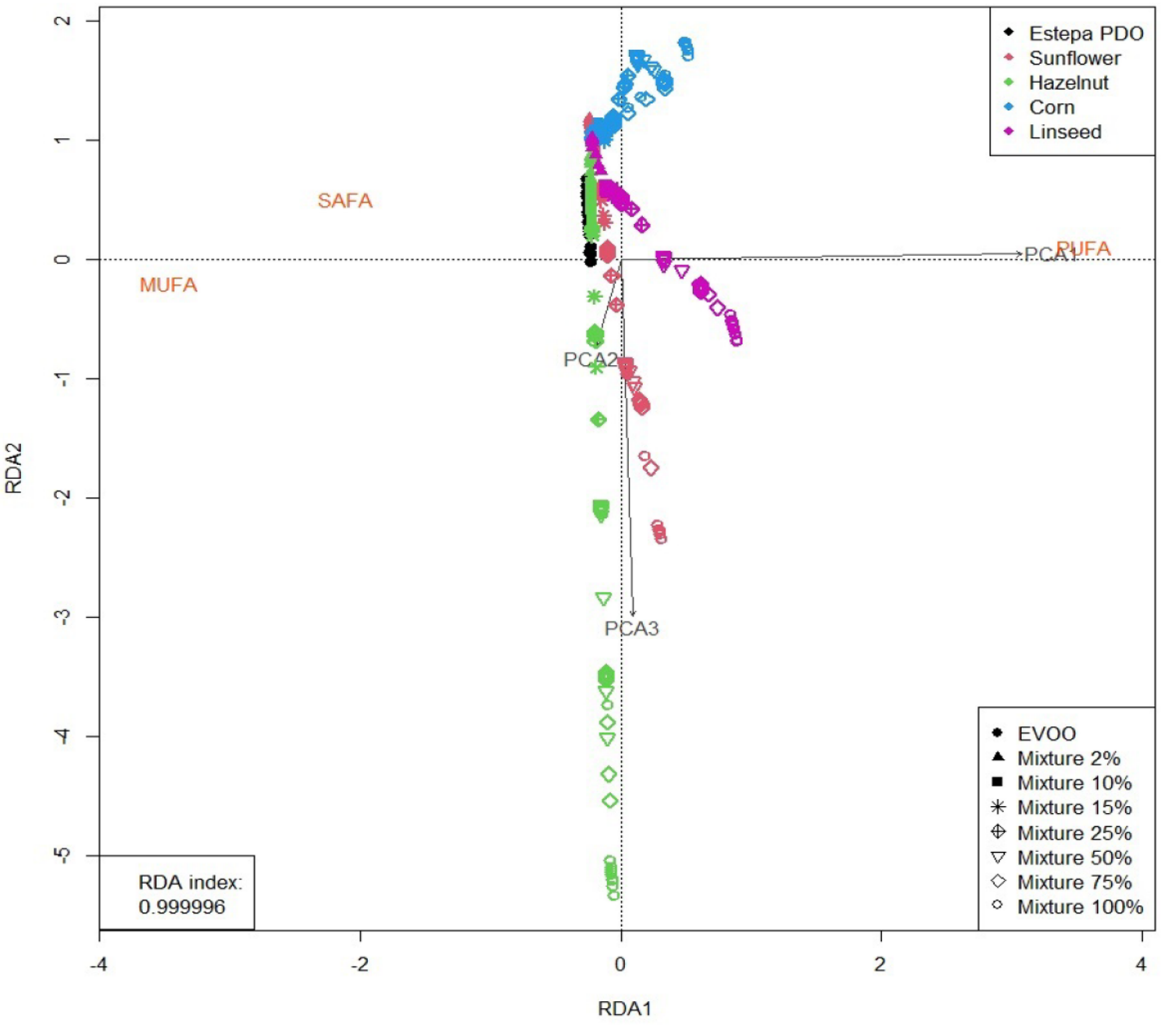
RDA representation from PCA factors.

As mentioned above, RDA is a constrained version of PCA, as it can reduce the high dimensionality of NIR spectra by taking into account the cause–effect relationship with the fatty acid profile obtained from GC, which is used as reference classical tecnique. Figure 2 shows the results of the application of RDA in the two-dimensional space formed by the first RDA components (RDA1 and RDA2, the ones explaining the highest percentage of variability of data) and containing (a) the fatty acid profile in SAFAs, MUFAs, and PUFAs (in orange); (b) the PCA factors summarizing the NIR spectral information (in gray); (c) the cases, using different colors for each oil (black, red, green, blue, and pink for Estepa PDO oil and mixtures with sunflower, hazelnut, corn, and linseed oils, respectively) and distinct symbols for the percentage of adulteration in mixtures (solid circles, solid triangles, solid squares, asterisks, crossed diamonds, open triangles, open diamonds, and open circles for EVOO and mixtures with 2, 10, 15, 25, 50, 75, and 100 percent adulteration, respectively). The RDA index is very close to 1, which indicates that the percentage of the total variance of **Y** (fatty acid content) explained by the two first RDA components is very close to 100% and thus highlights the excellent result in the goodness of fit of the procedure.

In the analysis of the RDA visualization of the data, the interpretation of the axes can be done by taking into account the points with the highest scores in absolute value. Besides, the proximity between cases and variables represents their association. Therefore, in this case, Figure 2 shows that RDA1 discriminates between oils as a function of their content in PUFA and SAFA–MUFA, while RDA2 to a lesser extent discriminates between the content of SAFA and MUFA. Thus, more in particular, (*i*) linseed oil (pink), corn oil (blue), and sunflower oil (red) show a high percentage in PUFA and a low percentage in MUFA; (*ii*) hazelnut oil (green) has a low percentage in SAFA and a high content in MUFA; and (*iii*) EVOO has a low percentage in PUFA and high content in MUFA and SAFA. These results are similar to the ones obtained in other studies.^36−38^

Moreover, RDA, considered as constrained form of PCA, provides a procedure for reducing the high-dimensional NIR spectral information, and the scores of cases in RDA1, RDA2, and RDA3 will be considered as explanatory variables in the following sections, where quantitative and qualitative models for authenticating vegetable oils are presented. Besides, the results will be compared with the ones obtained for the scores in PCA1, PCA2, and PCA3.

### Quantitative Analysis from PCA and RDA Factors: EVOO Percentage Prediction

**Figure 3:**
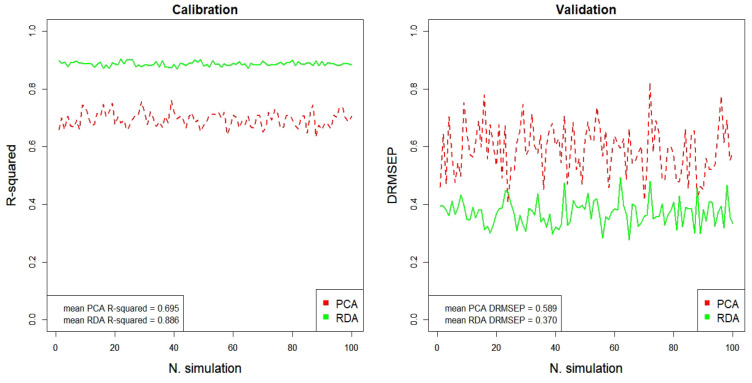
Cross-validated *R*^2^ and DRMSEP for EVOO percentage prediction from PCA and RDA factors.

This section evaluates regression models predicting the percentage of EVOO of every oil mixture and considering as explanatory variables the factors previously obtained by PCA and RDA analysis. The reliable estimation of this percentage is very important in the authentication of oils.^7^ The results of calibration (*R*^2^) and validation (DRMSEP) are presented in Figure 3, which simulates 100 different selections in the training and test subsets in order to cross-validate the results. Besides, the corresponding average values are included in the legends at the bottom left in the figures: *R*^2^ shows values closer to 1 and DRMSEP closer to 0 when RDA factors are used instead of PCA factors (see the solid green lines vs dashed red lines, respectively). Therefore, RDA factors are the best ones in the estimation of calibration and validation.

### Qualitative Analysis from PCA and RDA Factors: Classification of Oils

In this section, PCA and RDA factors are used as explanatory variables in machine learning classification models. First, the type of pure oil (distinguishing among Estepa PDO, sunflower, hazelnut, corn, and linseed oils) is considered as response variable. Then the classes of the dependent variable are defined, for every mixture of EVOO and another vegetable oil, as a function of the blend percentage (2, 10, 15, 25, 50, 75, 100%).

**Figure 4:**
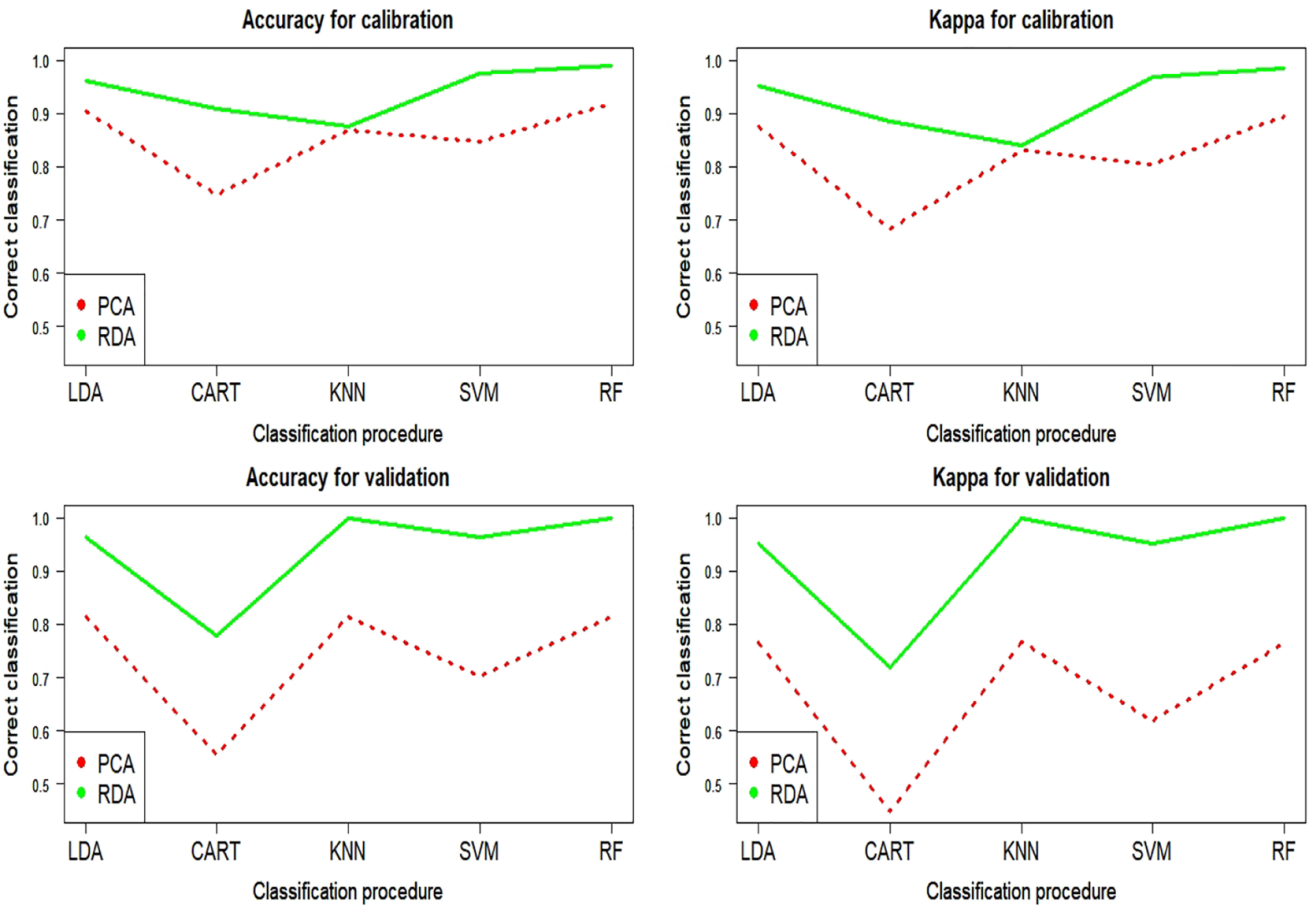
Accuracy and Kappa for calibration and validation in classification of pure oils from PCA and RDA factors and different algorithms.

Figure 4 compares the results in calibration and validation (*Y* axis) in the classification of the pure (not-blended) oils for the cases in which the NIR spectral information is summarized in PCA (dashed red lines) and RDA (solid green lines) factors. The machine learning classification algorithm (LDA, CART, KNN, SVM, or RF) is indicated on the *X* axis. The results are evaluated using accuracy and Kappa measures. The random number seed was reset before each iteration to ensure that the evaluation of each algorithm was performed using exactly the same calibration and validation subsets of data, so the results are directly comparable. Definitively, Figure 4 shows that the results in calibration and validation for accuracy and Kappa and every classification algorithm are better when RDA factors are used, as the correct classification rate is higher than the one obtained for PCA factors.

Subsequently, Tables 1–4 present the results on calibration and validation for every type of vegetable oil (sunflower, hazelnut, corn, and linseed oil) and percentage of mixture with EVOO. The table rows present the results for the different machine learning procedures (LDA, CART, KNN, SVM, and RF), and the table columns compare the percentages of correct classification obtained from PCA and RDA factors for each vegetable oil. In most of the classification algorithms, as marked by the up arrows, the results provided from RDA factors significantly improve the ones obtained from PCA factors in calibration and validation (for both accuracy and Kappa).

**Table 1:** Accuracy for Calibration in Classification of Blended Oils from PCA and RDA Factors and Different Algorithms^a^

	correct classification (%)											
	sunflower			hazelnut			corn			linseed		
	PCA	RDA	Dif	PCA	RDA	Dif	PCA	RDA	Dif	PCA	RDA	Dif
LDA	81.601	96.258	↑	76.823	80.788	↑	90.050	85.828	↓	74.151	95.364	↑
CART	59.222	75.359	↑	56.040	58.318	↑	47.681	76.192	↑	53.500	84.080	↑
KNN	82.677	94.591	↑	85.848	85.767	↓	91.348	86.828	↓	77.060	86.419	↑
SVM	69.505	95.348	↑	75.752	89.510	↑	90.070	94.313	↑	63.515	96.182	↑
RF	78.859	98.258	↑	85.843	92.328	↑	86.864	97.182	↑	70.333	98.182	↑

a Dif indicates ↑ or ↓ if the sign of RDA – PCA is positive or negative, respectively.

**Table 2:** Kappa for Calibration in Classification of Blended Oils from PCA and RDA Factors and Different Algorithms^a^

	correct classification (%)											
	sunflower			hazelnut			corn			linseed		
	PCA	RDA	Dif	PCA	RDA	Dif	PCA	RDA	Dif	PCA	RDA	Dif
LDA	78.248	95.532	↑	72.465	77.122	↑	88.214	83.241	↓	69.426	94.496	↑
CART	52.380	71.674	↑	49.132	51.321	↑	38.322	72.515	↑	46.350	81.602	↑
KNN	79.626	93.579	↑	83.259	83.055	↓	89.778	84.452	↓	72.912	83.759	↑
SVM	64.244	94.479	↑	71.317	87.625	↑	88.250	93.280	↑	56.965	95.458	↑
RF	74.972	97.927	↑	83.285	90.890	↑	84.426	96.645	↑	65.174	97.843	↑

a Dif indicates ↑ or ↓ if the sign of RDA – PCA is positive or negative, respectively.

**Table 3:** Accuracy for Validation in Classification of Blended Oils from PCA and RDA Factors and Different Algorithms^a^

	correct classification (%)											
	sunflower			hazelnut			corn			linseed		
	PCA	RDA	Dif	PCA	RDA	Dif	PCA	RDA	Dif	PCA	RDA	Dif
LDA	82.857	97.143	↑	80.000	74.286	↓	88.571	88.571	↑	80.000	100.00	↑
CART	42.857	51.429	↑	51.429	51.429	↑	37.143	51.429	↑	37.143	65.714	↑
KNN	80.000	88.571	↑	82.857	85.714	↑	85.714	85.714	↔	74.285	88.571	↑
SVM	80.000	94.286	↑	65.714	88.571	↑	80.000	91.429	↑	74.285	97.142	↑
RF	74.286	97.143	↑	82.857	88.571	↑	88.571	94.285	↑	71.428	100.00	↑

a Dif indicates ↑ or ↓ if the sign of RDA – PCA is positive or negative, respectively, and ↔ if RDA – PCA is zero.

**Table 4:** Kappa for Validation in Classification of Blended Oils from PCA and RDA Factors and Different Algorithms^a^

	correct classification (%)											
	sunflower			hazelnut			corn			linseed		
	PCA	RDA	Dif	PCA	RDA	Dif	PCA	RDA	Dif	PCA	RDA	Dif
LDA	79.904	96.651	↑	76.560	70.000	↓	86.577	86.667	↑	76.644	100.00	↑
CART	34.272	44.651	↑	44.392	44.651	↑	28.638	44.651	↑	28.704	60.747	↑
KNN	76.510	86.551	↑	79.923	83.333	↑	83.205	83.253	↑	70.000	86.564	↑
SVM	76.510	93.288	↑	59.801	86.641	↑	76.532	89.942	↑	69.914	96.647	↑
RF	69.856	96.650	↑	80.000	86.641	↑	86.577	93.320	↑	66.571	100.00	↑

a Dif indicates ↑ or ↓ if the sign of RDA – PCA is positive or negative, respectively.

## Conclusions

In this work, PCA was initially used to summarize the large NIR spectral information in some components describing a percentage of variability of data close to 100%. Then RDA, as a constrained version of PCA, was also considered to reduce the high-dimensional NIR spectral information. Later, both PCA and RDA factors were contemplated as explanatory variables in quantitative and qualitative estimation models useful to the authentication of EVOO. In particular, a regression model was considered to predict the numerical percentages of adulteration of mixtures of EVOO and other vegetable oils. The cross-validated results show that RDA factors provide better measures of calibration and validation than PCA factors. Besides, machine learning algorithms (i.e., LDA, CART, KNN, SVM, and RF) were used to classify in the different pure vegetables oils and, for a specific vegetable oil, in the class given by the percentage blended with EVOO. The results, measured by accuracy and Kappa for calibration and validation, also highlight the potential of RDA factors versus PCA ones. The reduction of NIR spectral information by using RDA factors represents a novelty in this field^13,14^ that even enables a percentage of correct classification near 100% for some machine learning algorithms and types of vegetable oil.

Finally, this work can be extended in some directions. First, recent works^39−41^ included relevant agroclimatic information, besides chemical spectral information, in the regression models to improve the estimation of the EVOO fatty acid profile. This aspect could improve the discrimination between pure and blended EVOO, as the redundancy analysis is based on the fatty acid profile. Second, a previous study^42^ achieved better predictions for the fatty acid content of oil from a functional approach for the chemical spectral information instead of the discretization treatment. Therefore, the functional version of RDA^43^ could also be proved in the classification of oils. To conclude, the Bayesian methods for NIR wavelet-based feature selection^44^ and the treatment of the fatty acid profile of oils as compositional data could also be investigated.^45^

## Supporting Information Available

The R code (JCIM-R-code.docx and JCIM-R-code.pdf) the data supporting the analyses (JCIM-R-data.zip) (ZIP)
The R code (JCIM_R code.R) and data files (in .txt format) (ZIP)

## Footnotes

a The proportion of the total variance of **Y** explained by each redundancy axis *k* (*k* = 1, ...,*g*) is given by the quotient . This proportion decreases from the first redundancy axis (with the highest proportion associated) to the last.

b These metrics have been selected as the usual default ones provided by R Project software to evaluate and compare models. However, other measures (e.g., sensitivity and specificity) were calculated and provided similar results.
